# Ferroptosis: a cell death connecting oxidative stress, inflammation and cardiovascular diseases

**DOI:** 10.1038/s41420-021-00579-w

**Published:** 2021-07-26

**Authors:** Yi Yu, Yuan Yan, Fanglin Niu, Yajun Wang, Xueyi Chen, Guodong Su, Yuru Liu, Xiling Zhao, Lu Qian, Ping Liu, Yuyan Xiong

**Affiliations:** 1 Key Laboratory of Resource Biology and Biotechnology in Western China, Ministry of Education, School of Medicine, Northwest University, Xi’an, 710069 Shaanxi China; 2 Department of Endocrinology, Xi’an No.3 Hospital, the Affiliated Hospital of Northwest University, Xi’an, Shaanxi 710018 P. R. China

**Keywords:** Necroptosis, Cardiovascular diseases

## Abstract

Ferroptosis, a recently identified and iron-dependent cell death, differs from other cell death such as apoptosis, necroptosis, pyroptosis, and autophagy-dependent cell death. This form of cell death does not exhibit typical morphological and biochemical characteristics, including cell shrinkage, mitochondrial fragmentation, nuclear condensation. The dysfunction of lipid peroxide clearance, the presence of redox-active iron as well as oxidation of polyunsaturated fatty acid (PUFA)-containing phospholipids are three essential features of ferroptosis. Iron metabolism and lipid peroxidation signaling are increasingly recognized as central mediators of ferroptosis. Ferroptosis plays an important role in the regulation of oxidative stress and inflammatory responses. Accumulating evidence suggests that ferroptosis is implicated in a variety of cardiovascular diseases such as atherosclerosis, stroke, ischemia-reperfusion injury, and heart failure, indicating that targeting ferroptosis will present a novel therapeutic approach against cardiovascular diseases. Here, we provide an overview of the features, process, function, and mechanisms of ferroptosis, and its increasingly connected relevance to oxidative stress, inflammation, and cardiovascular diseases.

Facts

- Ferroptosis as a novel form of cell death differs from other cell death such as apoptosis, necroptosis, pyroptosis and autophagy-dependent cell death.
- Ferroptosis is closely related to iron and lipid metabolism and triggered by various physiological conditions and pathological stresses in human beings and animals.
- Ferroptosis connecting oxidative stress and inflammation plays key roles in the pathogenesis of cardiovascular diseases such as atherosclerosis, stroke, ischemia-reperfusion injury and heart failure.

**Open questions**

- What are the additional drivers for lipid peroxidation or ferroptosis, and the subcellular location of lethal lipid peroxides?
- What is the mechanism of lipid peroxidation leading to cell death?
- Upon ferroptosis, what are the intracellular components released to promote inflammatory responses and neighbor cell death?
- What are the adverse effects of targeting ferroptosis in cardiovascular diseases?

## Introduction

Cardiovascular diseases (CVDs) are the leading causes of morbidity and mortality worldwide [1]. It is well-accepted that oxidative stress and inflammation play key roles in the pathogenesis of various cardiovascular disorders including atherosclerosis heart failure, ischemia/reperfusion injury and stroke, and the physiologic process of aging [2–4]. The imbalance between the production of reactive oxygen species (ROS) and the availability of antioxidants or free radical scavengers can cause oxidative stress, and that further activates an abundance of transcription factors and pro-inflammatory genes such as NF-κB, p53, HIF-1α, PPAR-γ, β-catenin/Wnt, and Nrf2 [5–7]. Activation of these transcription factors and genes recruit immune cells to secrete different cytokines and chemokines to trigger inflammation. Indeed, intracellular levels of ROS produced at a low level by normal aerobic metabolism are essential in normal cellular homeostasis and redox-dependent regulation of many signaling processes [8]. Whereas, a large number of ROS including superoxide (O_2_^•−^), hydroxyl radical (OH^•^), perhydroxyl radical (HO_2_•), and other types such as hydrogen peroxide (H_2_O_2_), hypochlorous acid (HOCl), and lipid peroxides (ROOH) [9], is generated as by-products of cellular aerobic metabolism upon different physiological and pathological changes, once exceeding the capacity of the cellular reductase mechanisms, which can result in direct or indirect functional damage to different molecules such as proteins, nucleic acids, and lipids, and eventually lead to cell death [10, 11].

Cell death is of essence in the process of development, homeostasis, and pathogenesis of acute and chronic diseases, which tends to be accompanied with inflammation dysregulation, cell dysfunction, and tissue damage [12]. Programmed cell death such as apoptosis, pyroptosis, and necroptosis play an important role in various physiological processes such as embryonic development, tissue homeostasis, and immunity in mammalian [13]. Ferroptosis is a new form of non-apoptotic cell death marked by the oxidative modification of phospholipid membranes via an iron-dependent mechanism [14]. Although the physiological function of ferroptosis is poorly understood, its implication in several human diseases such as tumorigenesis [15, 16], CVDs (e.g., atherosclerosis [17], ischemia-reperfusion injury (IRI) [18], cardiomyopathy [19, 20], and heart failure [21]) has been widely reported.

In recent years, accumulating studies demonstrate that ferroptosis is closely related to iron and lipid metabolism [22], which could be triggered by various physiological conditions and pathological stresses in human beings and animals [23]. As such, ferroptosis connecting oxidative stress and inflammation responses associated with iron and lipid metabolism plays a pathological role in CVDs. Here, we summarize the features, process, function, and mechanisms of ferroptosis, discuss its relevance with oxidative stress and cellular inflammation linking to cardiovascular disorders, raise the unsolved mysteries of ferroptosis, and provide new insights to consider the ferroptosis as the therapeutic target for the prevention and treatment of CVDs.

## Features and mechanisms of ferroptosis

### Features of ferroptosis

In 2012, ferroptosis was first termed by Stockwell Lab to define RAS-selective lethal small-molecule erastin-induced human fibrosarcoma cell death [23], which is iron-dependent, and morphologically and biochemically distinct from apoptosis, necrosis, autophagy-dependent cell death, and pyroptosis (Table 1). In ferroptotic cells, the intact cell membrane, cell swelling, reduction/disappearance of mitochondria crista, and absent nuclear condensation or chromatin margination are observed [24]. Biochemically, depleted intracellular glutathione (GSH) and inactivated activity of glutathione peroxidase 4 (GPX4) lead to cell ferroptosis, since overproduced lipid peroxides cannot be abolished by the GPX4-catalyzed reduction reaction.

**Table 1:** The main features of ferroptosis, apoptosis, necroptosis, pyroptosis, and autophagy-dependent cell death.

Cell death type	Ferroptosis	Apoptosis	Necroptosis	Pyroptosis	Autophagy-dependent cell death
Biological features	Inhibition of system X_c_^−^ and GPX4, cystine uptake reduction and GSH depletion, iron accumulation, and lipid peroxidation	Internucleosomal DNA fragmentation, activation of caspases, decreased mitochondrial membrane potential	Decrease in ATP, activation of RIP1, RIP3	Caspase 1-dependent processes and proinflammatory cytokine releases	Autophagosome formation and degradation
Morphological features	Cell swelling, electron-dense mitochondria, and outer-membrane rupture, reduction or vanishing of mitochondria crista	Plasma membrane blebbing, cell shrinkage, nuclear fragmentation	Rupture of the plasma membrane, cell swelling and disrupted cytoplasmic organelles, chromatin condensation	Plasma membrane rupture, organelle swelling, nuclear condensation	Formation of double membraned autolysosomes
Key regulators	ATP5G3, GPX4, NRF2, RPL8, RAS, SLC7A11	Bcl-2, bax, bak, caspase, p53, fas	RIPK1,RIPK3, MLKL	Caspase-1, gasdermins, NLRP3	mTOR, LC3, ATG5, Beclin 1
Inducers	Erastin, glutamate, SAS, lanperisone, SRS, RSL3, DPI7, FIN56, sorafenib, artemisinin	Apoptolidin A,hypoxia, FasL, staurosporine,UNC5B	TNF α, FasL, TWEAK	ZnO-NPs, Ivermectin	Rapamycin, simvastatin,thapsigargin valproate
Inhibitors	Ferrostatin-1, liproxstatin-1, desferoxamine, vitamin E, U0126, Trolox, DFO, CHX	NAIP, CTX1, c-IAP1/2, XIAP, ILP-2, survivin, Z-VADFMK	Necrostatin-1, NSA, kongensin-A,	Necrosulfonamide	Bafilomycin A1,3-MA, LY294002, wortmannin, Spautin-1,
Immune features	Pro-inflammatory	Mainly anti-inflammatory	Pro-inflammatory	Pro-inflammatory	Mainly anti-inflammatory

### The mechanisms of ferroptosis

Currently, the exact mechanism of ferroptosis is still elusive, although there are several hypotheses. The mechanism may refer to the formation of lipid pore, similar to the proteinaceous pores observed in necroptosis and pyroptosis [25]. It appears that extensive oxidation of polyunsaturated fatty acid (PUFA)-containing phospholipids might modify membrane structure and increase membrane permeability, eventually resulting in plasma membrane rupture in response to accumulation of lipid-ROS, e.g., lipid hydroperoxides. Lipid hydroperoxides may promote the production of reactive toxic aldehydes such as 4-hydroxynonenals (4-HNEs) and malondialdehydes (MDAs), which by crosslinking might inactivate essential cellular proteins to promote ferroptosis [16]. Several key regulators such as GPX4, iron, and ferroptosis suppressor protein 1 (FSP1) related signaling pathways were elucidated during the process of ferroptosis.

**Fig. 1:**
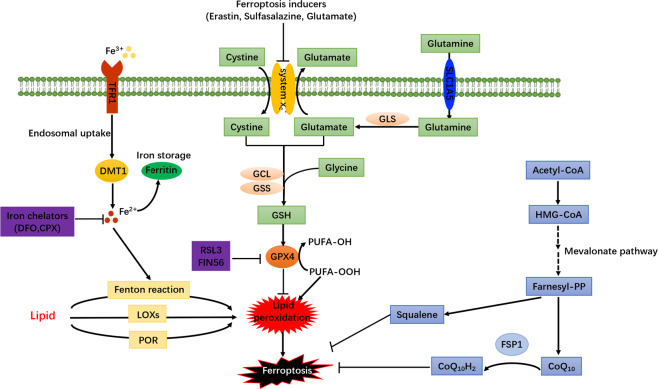
The regulatory pathways of ferroptosis. Ferroptosis is triggered by the excessive lipid peroxidation (LPO). Cysteine and reduced glutathione (GSH) availability are regulated by cystine-glutamate antiporter system X_c_^–^ or the transsulfuration pathway. The transsulfuration pathway is a metabolic pathway for the production of cysteine (Cys)-containing amino acids and the synthesis of GSH through the interconversion of cysteine and homocysteine. Glutamine can be transported into intracellular places by SLC1A5 transporter, and then converted into glutamate by glutaminase (GLS) to affect the GSH availability. GSH is a key cofactor of GPX4 for clearing lipid peroxides. The suppression of system X_c_^−^ by erastin or GPX4 activity by RSL3, which ultimately leads to cell ferroptosis. On the one hand, the peroxidation of PUFAs and excess irons is considered to be an important contributor. Iron uptake via the transferrin receptor-like TFR1 or degradation of ferritin iron stores enhances the labile iron pool, thereby cells are susceptible to ferroptosis via lipid hydroperoxides generation from the Fenton-like reaction. In addition, the mevalonate pathway is also involved in ferroptosis process by generating a series of biomolecules with potential anti-ferroptotic activity such as squalene, coenzyme Q10 (CoQ10), isopentenyl- pyrophosphate (IPP), and farnesyl pyrophosphate (FPP), which in part influences the ferroptosis occurrences. POR cytochrome P450 oxidoreductase, LOX lipoxygenase.

It is to note that apoptosis and necrosis inducers are incapable of triggering ferroptosis [26], suggesting that the mechanism underlying ferroptosis differs from that of apoptosis and necrosis. The ferroptosis inducers such as glutamate, erastin are able to drain GSH and inactivate the enzymatic activity of GPX4 via blocking the import of cystine by the cystine/glutamate antiporter (system X_c_^−^) [27, 28] (Fig. 1). GSH, as an important ferroptosis suppressor and non-enzymatic antioxidant, provides a vital defense system to protect cells from different types of oxidative stress [29]. The ratio between GSH and its oxidative status GSSG usually indicates the level of cellular oxidative stress. As a member of the glutathione peroxidases (GPXs) family, GPX4 utilizes GSH as the electron donor to reduce toxic lipid hydroperoxides (lipid-OOH) in cell membranes to non-toxic lipid alcohols (lipid-OH) and water. Lipid-OOHs are unstable, and can be broken down to reactive compounds like malonaldehyde, hexanal, and 4-hydroxynonenal, etc., which are capable of serving as “oxidative stress second messengers” because of their longed half-life and ability to diffuse from their formation site [30, 31]. From this point of view, the direct GPX4 inhibitor such as RSL3 could ultimately promote overwhelming lipid-ROS generation and induce ferroptosis. The cystine-glutamate antiporter system X_c_^−^ imports the extracellular cystine into the cytoplasm while it exports intracellular glutamate into the extracellular space. Cystine can be sequentially converted into GSH, which is indispensable in maintaining the intracellular redox balance as it can be utilized by GPX4 as a cofactor to alleviate lipid peroxidation (LPO) [32, 33] (Fig. 1). As a result, GSH deficiency, inactivation of GPX4 with ferroptosis inducer, or with the direct GPX4 inhibitor such as RSL3 ultimately promotes overwhelming lipid-ROS generation that leads to ferroptosis.

Ferroptosis is an iron-dependent cell death, and the normal inhibitors for apoptosis or necrosis can not prevent this process, but iron chelators work. Accumulated intracellular iron is another key to execute ferroptosis [14, 34] (Fig. 1). The hydroxyl radical (^•^OH) is the most reactive radicals that can be produced through the Fenton reaction and Haber-Weiss reaction from hydrogen peroxide by catalysis of metal species (iron, copper) [35, 36]. Extracellular Fe^3+^, binding with transferrin, can be imported into cytoplasm through transferrin receptor (TFR). Then intracellular Fe^3+^ form is transferred into the endosome and reduced to Fe^2+^ by iron oxide reductase STEAP3. The reduced form Fe^2+^ is transported into a labile iron pool from the endosome by the divalent metal transporter 1 (DMT1) [37] (Fig. 1). Excess Fe^2+^ in cells will initiate non-enzymatic LPO through Fenton reaction. Hydroxyl radical (•OH) directly generated from the Fenton reaction abstracts a hydrogen from PUFAs, forming a phospholipid carbon-centered radical (PL•), and proceeding with the oxygen addition to peroxyl radical (PL-OO•) to yield a phospholipid hydroperoxide (PL-OOH). Then accumulated lipid hydroperoxide is further converted to an alkoxyl radical (PL-O•) in the presence of ferrous iron, which subsequently reacts with adjacent PUFAs to initiate another lipid radical chain reaction [16]. Furthermore, iron is also involved in the enzyme-mediated peroxidation process by lipoxygenases (LOX) [38], a family of lipid-peroxidizing enzymes, that catalyzes the peroxidation of PUFAs to generate lipid peroxyl radical [39]. It is to note that both the cellular lipid hydroperoxides mediated by LOXs enzyme and autoxidized peroxyl radical-mediated LPO may contribute to initiate ferroptosis [38, 40]. Therefore, accumulated intracellular iron is a fuse to trigger ferroptosis.

Except for canonical glutathione-based GSH–GPX4 axis, FSP1-dependent pathway antagonizing ferroptosis has been recently uncovered. Bersuker et al. and Doll et al. coincidentally found that FSP1, previously known as apoptosis-inducing factor mitochondria-associated 2 (AIFM2), was an effective ferroptosis-resistance factor, and protected cells from GPX4 deletion-induced ferroptosis [41, 42]. Myristoylated FSP1 was recruited to the plasma membrane, and catalyzed the reduction of ubiquinone (CoQ_10_) by using NAD(P)H to form ubiquinol that functioned as a radical-trapping antioxidant to terminate LPO and finally suppressed ferroptosis. Thus, various biomolecules such as squalene and CoQ_10_ generated from mevalonate pathway also show potential roles in anti-ferroptosis [42–44] (Fig. 1). Nevertheless, a most recent study showed another potential mechanism of ferroptosis resistance for FSP1 independent of ubiquinol production that FSP1 modulates LPO through an ESCRT-III dependent membrane repair mechanism [45]. Moreover, beyond the role of tumor suppressor protein p53 in apoptosis, autophagy, and cell cycle, emerging evidence suggests that it sensitizes cells to ferroptosis via the regulation of cystine metabolism and ROS responses [15].

## Ferroptosis cross-linking ROS and inflammation

**Fig. 2:**
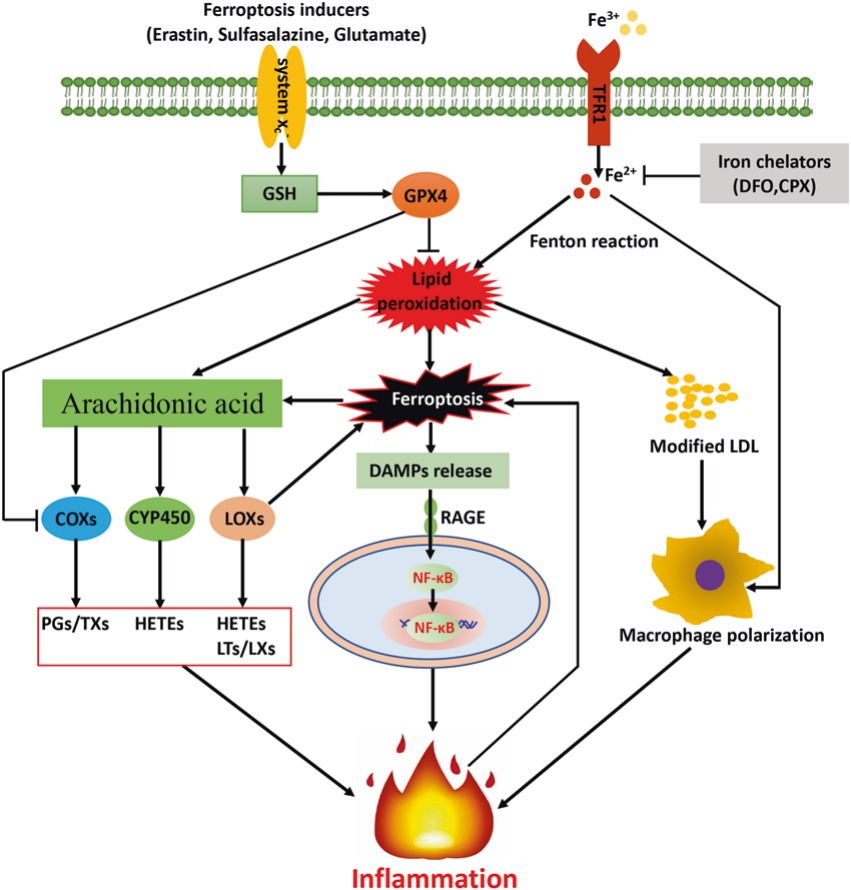
Cross-talk between ferroptosis and inflammation. ROS-induced oxidative stress exacerbates the production of lipid peroxidation (LPO). LPO-induced ferroptotic cell death results in the release of damage-associated molecular patterns (DAMPs) from dying cells, which may promote sterile inflammation through the receptor for advanced glycation end products (RAGE) to activate NF-κB. On the other hand, lipid peroxidation induced the increase in modified LDL partially contributes to inflammation activation via macrophage polarization. GPX4, the core regulator of ferroptosis, its activation suppressed ferroptosis and inflammatory responses via inhibiting arachidonic acid (AA) oxidation and NF-κB pathway activation. Moreover, GPX4 can up-regulate the expression of 12-lipoxygenase and cyclooxygenase 1 (COX1) that trigger inflammatory responses. AA, an unsaturated fatty acid and released from the cell membrane in response to various cytokines, peptides, and growth factors, that can be metabolized by cyclooxygenases (COXs), lipoxygenase (LOX), and cytochrome P450 (CYP450) monooxygenases to synthesize bioactive proinflammatory mediators such as prostaglandins (PGs), leukotrienes (LTs), lipoxins (LXs), hydroxyeicosatetraenoic acids (HETE) and hydroperoxyeicosatetraenoic acids (HPETE) that promote inflammatory responses. Finally, another vital regulator of ferroptosis, iron also participates the regulation of immune system via modulating M1 macrophage polarization.

Various types of regulated cell death (RCD) are implicated in the regulation of inflammation. In recent years, a concept of “necroinflammation” was raised and defined to describe the inflammatory response to necrotic cell death in a living organism [46, 47]. As a consequence of cell death, its intracellular components are released as pro-inflammatory damage-associated molecular patterns (DAMPs) such as ATP, nucleotides, histones, and high-mobility group protein B1 (HMGB1) and pro-inflammatory cytokines, such as interleukin‑1α (IL‑1α), IL‑33, and interferon‑γ (IFNγ) upon plasma membrane rupture, which triggers the innate immune system [48]. Following an initial event of RCD, DAMPs accumulation can trigger tissue inflammation which can further promote RCD to form a self-amplified circuit of necro-inflammatory loop that leads to exaggerated cell death and inflammation, eventually resulting in tissue damage and organ dysfunction [48, 49]. Unlike the in-depth understood roles of immunologically non-silent types of regulated necrotic cell death such as necroptosis and pyroptosis, the study of necroinflammation in ferroptosis is still in the early stages [50]. Ferroptotic cell death is also capable of releasing DAMPs that promote sterile inflammation and the development of numerous inflammatory diseases, for example, via its receptor for advanced glycation end-products (RAGE) to activate NF-κB [51, 52] (Fig. 2).

In addition, inflammation is closely associated with oxidative stress, which are able to trigger a series of transcription factors such as Nrf2, NF-κB1, and pro-inflammatory cytokines such as TNF-α, resulting in the differential expression of inflammatory cytokines, chemokines, and anti-inflammatory molecules [7, 53]. Redundant ROS induces oxidative stress and consume intracellular antioxidants, which further exacerbates the production of LPO and inflammatory responses in a vicious circle. Moreover, LPO drives the increase in modified LDL, which in part promotes inflammation via macrophage polarization (Fig. 2). Therefore, LPO can be observed in multiple physiological conditions such as cell death and inflammatory response in the pathophysiology of various diseases.

GSH and GPX4, the key regulators of ferroptosis, are also crucial in mediating inflammatory responses. GSH, as the most abundant antioxidant, is necessary to buffer rising ROS and prevent inflammation-associated cellular damage [54]. Disruption of GPX4 in a human carcinoma cell line can up-regulate the expression of 12-lipoxygenase and cyclooxygenase 1 (COX1) that triggers inflammatory responses [55, 56]. On the contrary, GPX4 activation suppressed ferroptosis and inflammatory responses via blunting arachidonic acid (AA) oxidation and NF-κB pathway activation, accompanying by decreased ROS generation [57]. AA, an unsaturated fatty acid, released from the cell membrane in response to various cytokines, peptides and growth factors [58], can be metabolized by COX, LOX, and cytochrome P450 (CYP450) monooxygenases to synthesize bioactive inflammatory mediators such as prostaglandins (PGs), leukotrienes (LTs), epoxyeicosatrienenoic acids (EETs) and hydroxyeicosatetraenoic acids (HETEs) [59] (Fig. 2), which play important roles in inflammatory responses and many physiological processes [60–62]. LOXs have been demonstrated as central players in ferroptosis-associated accumulation of the products of LOX catalysis [38].

Iron, a vital regulator of ferroptosis, also participates in the regulation of immune system. Iron metabolism in inflammation has been well characterized and intracellular iron overload promotes M1 macrophage polarization [63]. Handa et al. reported that dietary iron overload in mice was able to induce hepatic macrophage M1 activation, and was also accompanied by hepatic fibrogenesis and steatohepatitis [64]. DIBI, a novel iron-chelator, was found to reduce the levels of inflammatory mediators and restore functional capillary density in the intestinal muscle layer for sepsis treatment [65, 66]. Interestingly, iron was found to alter the balance between M1/M2 macrophage polarization, leading to macrophage-driven inflammation and fibrogenesis in nonalcoholic fatty liver disease (NAFLD) [64].

## Ferroptosis in CVDs

### Ferroptosis in atherosclerosis

In the past decade, several cell death types such as apoptosis, pyroptosis, necroptosis, and ferroptosis, are reported to contribute to atherosclerotic lesions formation during all stages. Liu et al. and our group reported that macrophage and VMSCs apoptosis is necessary to reduce macrophage burden and stabilize the atherosclerotic plaque within the developing lesion in Apo E^−/−^ mice [67, 68]. In addition to apoptosis, pyroptosis and necroptosis are also implicated in advanced atherosclerotic lesions and ruptured human plaques [69]. In Apo E^−/−^ mice, inhibition of pyroptosis and necroptosis is able to ameliorate atherogenesis and reduce necrotic core size [70–72]. Ferroptosis, a newly discovered form of iron and LPO-dependent cell death, has been also implicated in atherogenesis. Bai et al. showed that inhibition of ferroptosis by ferrostatin-1 (Fer-1) administration alleviates high-fat diet (HFD)-induced atherosclerosis in Apo E^−/−^ mice through reducing LPO in mouse aortic endothelial cells [17].

**Fig. 3:**
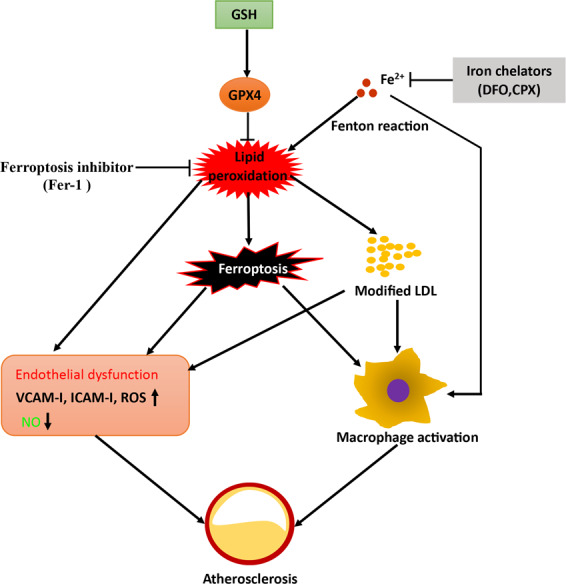
The role of ferroptosis in atherosclerosis. Atherosclerosis is a chronic inflammatory disorder. Ferroptosis in endothelial cells and macrophages can lead to endothelial dysfunction (e.g., VCAM-I, ICAM-I, ROS increase, NO decreases) and macrophage activation, which contributes to the generation of atherosclerosis. Ferroptosis-associated peroxidation of LDL in the endothelium is capable of causing endothelial dysfunction and macrophages activation. The PUFA-OOH, the main source of lipid peroxidation (LPO) may also trigger endothelial ROS increase, nitric oxide (NO) decrease, chronic inflammation in macrophages and formation of foam cells. Iron-catalyzed free radical reactions cause not only ferroptosis but also the oxidation of LDL in endothelial cells, smooth muscle cells or macrophages, which are also involved in the formation of atherosclerotic lesions.

The PUFA-OOH, the main source of LPO, may trigger endothelial ROS increase, nitric oxide (NO) decrease, chronic inflammation in macrophages, and formation of foam cells, and eventually contributes to the formation of atherosclerotic lesions. Besides, ferroptosis-associated peroxidation of LDL in the endothelium is capable of causing endothelial dysfunction and macrophages activation. Prior to discovery of ferroptosis, overexpression of GPX4 was reported to scavenge extra ROS and phospholipid hydroperoxides, and significantly slow down the progression of atherosclerotic plaque in Apo E^−/−^ mice [73]. Apart from the GPX4, extensive studies have shown that iron was also involved in the development of atherosclerotic lesion through affecting LPO in vitro and in vivo [74, 75]. Iron-catalyzed free radical reactions cause oxidation of LDL in endothelial cells, smooth muscle cells, or macrophages, which may be risk factors in the formation of atherosclerotic lesions [76]. Oxidized-low density lipoprotein (Ox-LDL) induced ferroptosis and iron accumulation were observed in mouse aorta endothelial cells, and this event can be reversed by ferroptosis inhibitors [17]. Transferrin, the major plasma iron-binding molecule, interacts with transferrin receptor protein 1 (TFR1) to deliver extracellular Fe^3+^ into cells, leading to iron pool overload and increased cell susceptibility to ferroptosis [77]. Ferritin and LDL-cholesterol levels showed a synergistic association with the incidence of CVD and death [78]. As LPO as well as iron deposition are prominent features of atherosclerotic plaques [79], it is rational to deduce that ferroptosis in ECs, VMSCs, and macrophages may be implicated in the progression of atherosclerotic plaque destabilization. Taken together, ferroptosis connecting LPO, inflammation, and iron storage may play a crucial role in the progression of atherosclerosis (Fig. 3).

### Ferroptosis in ischemia/reperfusion injury

IRI refers to the tissue damage caused by blood supply restores to ischemic tissue (reperfusion) after a period of ischemia. Reperfusion of organ is indispensable event to rescue tissue from the long-term oxygen deprivation. However, restoration of circulation is accompanied with inevitable cell death, oxidative stress damage, inflammation and programmed cell death, and eventually results in tissue injury such as myocardial infarction (MI) [80]. As such, IRI is a distressing issue at the beginning of organ transplantation era and impedes the recovery of organ transplanted patient. During the process of ischemic tissue restoring to aerobic metabolism, free radicals might be generated via oxidizing various cellular components [81]. Garci-Gil et al. have shown that LPO is the major contributor to cause ischemia-reperfusion oxidative injury on a pig pancreas transplantation model [82]. Another study reported that reperfusion of ischemic cardiac tissue enhanced the concentration of LPO product 4-HNE, contributing to the declines in mitochondrial function [83]. In 2013, Linkermann et al. found combined inhibition of mitochondrial permeability transition (MPT)-induced regulated necrosis (RN) and receptor-interacting protein kinase (RIPK)1-mediated necroptosis offered protective effects in IRI model [84]. Further study based on an artificial model of GPX4 deletion in the proximal tubules indicated that knockout of GPX4 caused cell death in a pathologically relevant form of ferroptosis and spontaneous tubular necrosis, and the inhibition of ferroptosis by liproxstatin-1 was able to mitigate ischemia/reperfusion-induced tissue damage [85]. Moreover, a variety of studies have shown that ferroptosis is involved in IRI in the liver [86], brain [87], and heart [88]. More recently, several studies have uncovered some interesting finding that inhibition of ferroptosis ameliorated IRI in different tissues. Inhibition of ferroptosis by ferrostatin-1 (Fer-1) was shown to reduce infarct size, improve left ventricular (LV) systolic function, and attenuate LV remodeling in myocardial IRI via lowering the levels of pro-ferroptotic LPO and reducing cardiomyocyte cell death, providing evidence that ferroptosis regulates cardiomyocyte cell death in the context of MI [89]. In addition, a single cell undergoing NADPH-depleting ferroptosis may deplete the NADPH concentration of neighbor cells that are connected to the cytoplasm of this cell, which causes the synchronized necrosis of neighbor cells in a functional organ [90]. As this phenomenon of synchronized RN is predominantly mediated by ferroptosis, IRI may critically involve the spreading of necrotic lesions within functional units such as a tubule or maybe between cardiomyocytes [91].

**Fig. 4:**
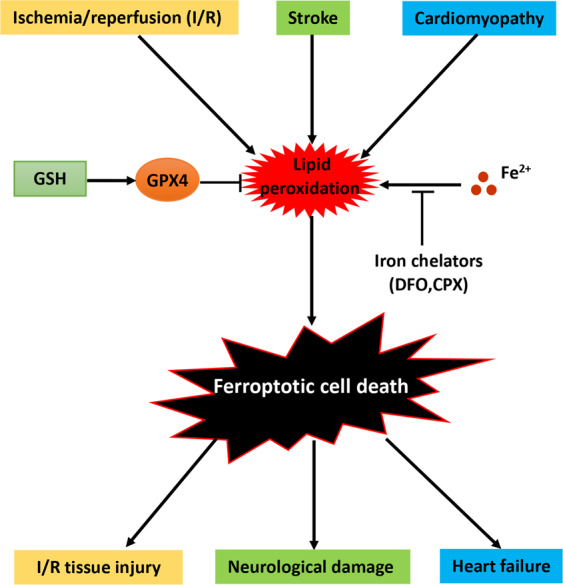
The role of ferroptosis in I/R injury, stroke and heart failure. Ischemia-reperfusion (IR), stroke, and cardiomyopathy are accompanied with not only inevitable mitochondrial dysfunction, lipid peroxidation (LPO) and inflammation but also ferroptotic cell death, which results in tissue injuries such as ischemia-reperfusion injury, neurological damage, and heart failure that can be ameliorated by inhibition of ferroptosis via increasing GSH levels, GPX activity or iron chelation.

Iron overload is another factor to promote tissue oxidation during ischemia and reperfusion, and becomes a novel risk factor for predicting hepatic I/R injury in liver transplantation and lung oxidative injury with ischemia/reperfusion in rat lungs [18, 92–94]. As such, iron-dependent ferroptosis is doomed to affect the process of IRI (Fig. 4). Yamada et al. found that I/R was able to lead to liver damage, LPO as well as upregulation of the ferroptosis marker Ptgs2 in a murine model, and all of these effects were significantly alleviated by ferroptosis-specific inhibitor ferrostatin-1 and iron chelator deferoxamine [18]. In addition, tissue ischemia and reperfusion are accompanied with pro-inflammation, thus ferroptosis-associated inflammatory responses may play an unexpected role in IRI. Collectively, ferroptosis contributes to the development of tissue I/R induced injury (Fig. 4), and presents a potential therapeutic target for the prevention of organ I/R damage, e.g., solid organ transplantation.

### Ferroptosis in stroke

Stroke is one major cause of death worldwide, and its incidence including ischemia or hemorrhage leads to the interruption or reduction of body blood supply to brain. Ferroptosis has been shown to be implicated in the pathological cell death associated with stroke [27]. Alim et al. reported that selenium suppresses ferroptotic cell death to protect neurons from hemorrhagic or ischemic stroke damage by activating the transcription factors TFAP2c and Sp1 [95]. Iron has an fundamental role in many physiological processes, including erythropoiesis, immunity, and oxidative metabolism [96]. Iron is also a key component of cytochromes a–c of the oxidative chain, and responsible for the production of adenosine triphosphate (ATP) [97]. However, disturbances in iron homeostasis have been linked to neuronal damage following ischemic injury [98]. By catalyzing Fenton reactions that convert superoxide and hydrogen peroxide into reactive toxic hydroxyl radicals, iron can induce ferroptosis and subsequent neuronal injury [99]. As such, iron is causally linked to LPO production such as lipid hydroperoxides that promotes ferroptosis, and to conditions that predispose to ischemic stroke [100] (Fig. 4). Both basic and clinical evidence validate that LPO dysregulation mediates stroke injury. LPO products such as MDA, thiobarbituric acid-reactive substances and 4-hydroxynonenal already showed the obvious augmentation and detrimental effects in ischemic stroke [101–103]. Clinical study also exhibited that the mean values of lipid hydroperoxidesin in the plasma of stroke patients were ~2.5-fold higher than controls [104]. Moreover, the rupture of the blood vessel wall in hemorrhagic stroke results in the accumulation of hemoglobin and heme iron at the extracellular milieu and subsequent iron-induced LPO and ferroptosis, thereby furtherly exacerbating the stroke-induced brain injury.

### Ferroptosis in heart failure

Heart failure occurs when the ability of heart pumping blood to the rest of our body is seriously compromised. Cardiomyopathy is a major factor to lead to heart failure. The loss of terminally differentiated cardiomyocytes is a lethal pathogenic factor in the pathophysiological process of cardiomyopathy. Whereas, the molecular mechanisms of cardiomyocyte death are still not well elucidated.

It was demonstrated that ferroptosis is highly correlated with cardiomyocyte death, and mammalian target of rapamycin (mTOR) plays an important role in protecting cardiomyocytes against excess iron and ferroptosis by inhibiting the production of lipid-derived ROS [105]. In the most recent study, Wang et al. group presented that iron overload-induced ferroptosis was a major contributor to doxorubicin (DOX) induced cardiomyopathy, and treatment with the ferroptosis inhibitor ferrostatin-1 remarkably improved the survival of DOX-treated mice. Moreover, they figured out that heme oxygenase-1 (HMOX1) was the major culprit for iron release in DOX-induced cardiotoxicity and provided intriguing insights for the first time into the role of ferroptosis in cardiac cell death linked to cardiomyopathy [19, 106]. Next, Wang et al. further revealed that ferrostatin-1adminstration rescued high-iron diet-induced severe cardiac injury and hypertrophic cardiomyopathy, with molecular features typical of ferroptosis, including reduced GSH levels and increased LPO. Finally, they found that the expression of ferroptosis regulator SLC7A11 transporting cystine (the precursor of GSH) into the cytosol was suppressed in conditional Fth knockout cardiomyocytes, and overexpressing SLC7A11 in cardiomyocytes selectively increased the levels of GSH that prevented cardiac ferroptosis [20]. Puerarin, one of the most abundant phytoestrogens with antioxidant activity, could improve myocyte loss during heart failure induced by pressure overload through ferroptosis suppression in rat model [21]. In addition, the classical ferroptosis inducer erastin was also reported to damage cardiomyocytes by initiating ferroptosis, while ENPP2, a lipid kinase generating the lipid mediator lysophosphatidic acid, could protect cardiomyocytes from the erastin-induced ferroptosis by decreasing ROS generation [107]. Moreover, inhibition of endogenous expression of TLR4 and NADPH oxidase 4 that were upregulated in rat cardiac tissue after heart failure, showed heart failure relief by suppressing ferroptosis in cardiac cells [108]. These studies disclose ferroptotic cell death is implicated in the regulation of heart failure (Fig. 4). However, the molecular mechanisms of ferroptosis in cardiomyocytes still require further investigation.

## Therapeutic strategy targeting ferroptosis in CVDs

Ferroptosis connecting oxidative stress, inflammation, and cell death, plays a pathophysiological role in CVDs. Therefore, trapping ferroptosis is promising strategy to treat ferroptosis-associated cardio- and vascular- disorders. To study the potential role of ferroptosis in vivo, effective and specific small molecular ferroptosis inhibitors have been identified and can be categorized into iron chelators, lipophilic radical-trapping antioxidants, and system X_c_^–^ [109].

### Iron chelators

As iron participates in the generation of lipid-ROS and ferroptosis occurrence [110], the medical use of chelators in humans in the prevention of ferroptosis-associated CVDs (e.g., atherosclerosis, IRI and cardiomyopathy) is optimistic [111]. Deferoxamine (DFO) is a clinically approved iron chelator with a very high affinity to bind with the trivalent ferric ion to remove iron [111]. Various studies showed that DFO reduced cytosolic ROS production and ferroptotic cell death [26]. In vivo, DFO also shows cardioprotective effects by inducing ischemic preconditioning-like effects in I/R models, and reduction of mice atherosclerotic lesion by inhibiting inflammation and impairing nitric oxide action [112–114]. In type 2 diabetic rat model, inhibition of ferroptosis by DFO treatment may prevent post-stroke cognitive impairment [115]. DFO administration showed cardioprotective effects in I/R models via inhibiting cytosolic ROS production and ferroptotic cell death [112]. Dexrazoxane (DXZ) is the only iron chelator drug approved by the FDA and shows cardioprotective effects with ferroptosis inhibition in animal models of doxorubicin-induced cardiomyopathy [19]. The clinical use of iron chelators in treating iron load disorders makes it a potential therapeutical strategy in the prevention of ferroptosis-related CVDs.

### GPX4 and GSH

GPX4 is critical in preventing excess free radical generation and ferroptotic cell death [116], as it utilizes GSH as the electron donor to reduce toxic lipid hydroperoxides in cell membranes to the corresponding alcohols and water [117]. Ferroptosis is associated with GPX4 inactivation that leads to lipid oxidative stress. Conditional GPX4 knockout in myeloid lineage cells results in LPO-dependent caspase-11 activation and increase of N-terminal gasdermin D (GSDMD) fragments, which triggers macrophage pyroptotic cell death and release of DAMPs such as high-mobility group box 1, alarmin and pro-IL-1β [118]. Taken together, it is worth to notice that the phenomenon of synchronized regulated pyroptosis might occur and be mediated by GPX4-dependent ferroptosis. Alternatively, GPX4 depletion initially triggers pyroptotsis and subsequently regulates inflammation and ferroptosis. In vivo, GPX4 deficiency in mice caused embryonic lethality, which may be correlated with increased ferroptotic cell death and enhanced LPO [119, 120].

In addition, an adequate supply of GSH is beneficial to attenuate ferroptosis. Thiol supplementation with GSH selectively improves human endothelial dysfunction by enhancing NO activity [121]. While mitochondria-specific transgenic overexpression of GPX4 in mitochondria is also able to decrease LPO and attenuate rat I/R injury [122]. Given GPX4 activity and GSH level that could modulate oxidative stress, thus any dysregulation of GSH and GPX4 might promote ferroptosis and lead to adverse cardiac disorders. Several nature products with antioxidant activity such as vitamins and vitamin precursors have shown efficient inhibitory effects of ferroptotic cell death and potential cardioprotective effects [116, 123]. Vitamin E supplements protect against atherosclerosis in vivo, this action might be through preventing ferroptosis by lowering the oxidation of LDL [124, 125].

### Ferrostatins and liproxstatins

Ferrostatins and liproxstatins are screened compounds that act as lipophilic antioxidants to inhibit LPO associated with GPX4 deficiency or inhibition induced ferroptosis. Ferrostatin-1 (Fer-1), one of the ferrostatin family, is the extensively researched compound that suppresses different inducers induced ferroptosis in a variety of cell lines, tissue types, and disease models [22]. At the most recent study, Bai et al. showed Fer-1 ameliorated HFD-induced AS lesion in Apo E^–/–^ mice with the reduction of iron accumulation and LPO, and increase of expressions SLC7A11 and GPX4. Oxidized-low density lipoprotein (ox-LDL)-induced mouse aortic endothelial cells dysfunction can be significantly improved by Fer-1 treatment [17]. Moreover, Fer-1 also protects the heart against I/R injury and doxorubicin-induced cardiomyopathy by inhibiting ferroptosis [19]. Not only that, the administration of Fer-1 in vivo reduced neuronal death and improved neurologic function after intracerebral hemorrhage (ICH) by inhibiting lipid ROS generation and COX-2 expression [87]. Liproxstatin-1 (Lip-1), the first identified molecule of liproxstatin class, suppresses ferroptosis by scavenging free radical to prevent PUFA oxidation [85, 126]. Lip-1 treatment showed remarkable reduction in myocardial infarct sizes and maintained mitochondrial structural integrity and function against I/R injury by reducing VDAC1 levels and restoring GPX4 levels [127]. Therefore, screening a variety of small-molecule as ferroptosis inhibitors like Fer-1 and Lip-1 could be optimistic strategy for CVD drugs development in the future.

### System X_c_^–^

System X_c_^–^, a crucial negative regulator of ferroptosis, structurally consists of the twelve-pass transmembrane transport protein cystine/glutamate transporter (SLC7A11) and the single-pass transmembrane regulator protein 4F2 cell-surface antigen heavy chain (SLC3A2), and is responsible for maintaining redox homeostasis by importing cystine to synthesize GSH [128, 129]. Inhibiting system X_c_^–^ with compounds such as sorafenib and sulfasalazine can promote ferroptosis, while overexpression of system X_c_^–^ components by contrast is capable of diminishing erastin-induced ferroptosis [23]. Fang et al. showed conditional Fth-deficient cardiomyocytes suppressed the expression of the ferroptosis regulator SLC7A11, and overexpressing SLC7A11 selectively in cardiomyocytes increased GSH levels and suppressed cardiac ferroptosis, indicating that targeting SLC7A11 might offer new therapeutic opportunities for treating and/or preventing cardiac complications [20].

## Potential risk of ferroptosis-inducing agents to cardiovascular system

Over the past a few years, emerging evidence indicates that induction of ferroptotic cell death by ferroptosis-inducing agents in cancer cells may provide a potent strategy for cancer therapy. Some ferroptosis-inducing (FIN) compounds from improved analogs of erastin with increased solubility and selectivity showed potency in suppressing tumor growth in xenograft mouse tumor models [130]. Sorafenib, a multiple kinase inhibitor and FDA approved drug, was revealed to induce ferroptosis and exhibit anti-tumor effects in various cancer cell lines such as hepatocellular carcinoma cells, human kidney cancer cells and non‑small cell lung cancer [131, 132]. Whereas, the high risk for cardiovascular complications should be aware when these ferroptosis-inducing agents are clinically utilized in cancer treatment. Duran et al. showed that sorafenib can cause cardiotoxicity through inducing myocyte necrosis, which dramatically increases mortality in the setting of MI [133]. A clinical study reported that the use of sorafenib for advanced renal cell carcinoma induced acute MI [134]. Moreover, sorafenib-associated heart failure was reported in treatment of advanced stage hepatocellular carcinoma [135]. Currently, the molecular mechanisms of FIN agents posing a potential risk to cardiovascular system still remain elusive and require further investigation. Likely, the induction of ferroptosis by FIN agents may promote the synchronized RN and inflammation reaction, resulting in cardiovascular cells dysfunction. Therefore, the treatment of cancers based on ferroptosis induction should be cautious to reduce damage to the cardiovascular system.

## Conclusions and future perspectives

In recent years, studies on the role of ferroptosis in various diseases such as cancer and CVDs receive extensive attention. PUFA or LPO and excessive iron accumulation are obvious features of ferroptosis, that cannot be observed in other forms of cell death. Moreover, ferroptosis is tightly associated with various biological processes, including amino acid, iron, and PUFA metabolism, and the biosynthesis of glutathione, phospholipids, and coenzyme Q10 [27]. Therefore, ferroptosis as a nexus linking oxidative stress and inflammation is inevitable to play important roles in the pathogenesis of CVDs such as atherosclerosis, stoke, IRI, and heart failure. Currently, some approved and safe molecules that regulate ferroptosis by directly or indirectly targeting iron metabolism and LPO are already used to ameliorate cardiovascular injury, doxorubicin cardiotoxicity, and other diseases [111, 112]. Cardiac pathological conditions such as acute myocardial infarct (AMI) and I/R injury are closely bound up with ROS overproduction and further influence ferroptosis [136]. Ferroptosis studies in heart diseases validate existing mechanisms, suggesting that disturbance of ferroptosis might be beneficial to alleviate myocardial cell death and dysfunction in diseases like iron overload cardiomyopathy and I/R injury.

Collectively, ferroptosis might be a sensor in response to the pathophysiological alterations that disrupt the iron, ROS and inflammation homeostasis of our cardiovascular system. The study of ferroptosis is still undergoing and there are many unsolved mysteries such as the drivers of LPO, the subcellular location of lethal lipid peroxides and the exact mechanism of LPO leading to cell death [137]. Answers to these questions will provide novel insights into the mechanisms of ferroptotic cell death and associated human diseases, as well as new therapeutic strategies for CVDs treatment.
